# Short‐ and mid‐term effects of empagliflozin on sodium balance and fluid regulation in chronic heart failure

**DOI:** 10.1002/ejhf.70078

**Published:** 2025-11-04

**Authors:** Venera Bytyqi, Dennis Kannenkeril, Julie Kolwelter, Peter Linz, Agnes Bosch, Kristina Striepe, Marina V. Karg, Armin M. Nagel, Michael Uder, Mario Schiffer, Stephan Achenbach, Roland E. Schmieder

**Affiliations:** ^1^ Department of Nephrology and Hypertension University Hospital Erlangen, Friedrich‐Alexander University Erlangen‐Nuremberg (FAU) Erlangen Germany; ^2^ Department of Cardiology University Hospital Erlangen, Friedrich‐Alexander University Erlangen‐Nuremberg (FAU) Erlangen Germany; ^3^ Institute of Radiology, University Hospital Erlangen, Friedrich‐Alexander University Erlangen‐Nürnberg (FAU) Erlangen Germany

**Keywords:** Empagliflozin, Chronic heart failure, Sodium balance, Fluid regulation, Renal adaptation

## Abstract

**Aims:**

Sodium–glucose co‐transporter 2 inhibitors have become a cornerstone in managing chronic heart failure (CHF). While their acute impact on urinary glucose and sodium excretion is well‐established, their mid‐ and long‐term persistence of these effects remains uncertain. This study investigated fluid and sodium balance over 3 months in a randomized, placebo‐controlled trial (NCT03128528).

**Methods and results:**

Overall, 74 patients with New York Heart Association class II–III CHF and an ejection fraction (EF) ≤49% were randomized (2:1) to empagliflozin 10 mg (*n* = 48) or placebo (*n* = 26). Sodium, potassium, glucose, urea, and urine were determined from standardized 24‐h urine collections. Free water clearance (FWC) and plasma/urine osmolality were calculated. Body weight was measured, and dedicated sodium magnetic resonance imaging (^23^Na‐magnetic resonance imaging) was performed to quantify skin and muscle sodium levels at baseline, at 1 month, and at 3 months. Patients (mean age 66.4 years; 84% male; EF 40%; baseline N‐terminal pro‐B‐type natriuretic peptide 707.9 pg/ml) were followed up at 1 and 3 months. Empagliflozin significantly increased natriuresis at 1 month (*p* = 0.040), while natriuresis returned to baseline by 3 months. Skin sodium content decreased at 1 month (*p* = 0.039) and remained reduced at 3 months (*p* = 0.013), while muscle sodium was unchanged. Persistent glucosuria (*p* < 0.001) increased urine osmolality at 3 months (*p* = 0.003). Urine volume increased transiently at 1 month (*p* = 0.046) but normalized by 3 months. Empagliflozin‐treated patients showed a reduction in FWC at 1 and 3 months (*p* < 0.001), with a compensatory rise in copeptin levels, indicating increased vasopressin activity (1 month: *p* = 0.020; 3 months: *p* = 0.001).

**Conclusions:**

Mid‐range effects of empagliflozin in heart failure with reduced EF include transient natriuresis and sustained glucosuria, with compensatory reductions in FWC. Reductions in skin sodium content were maintained, and volume homeostasis in CHF patients stabilized after 3 months.

## Introduction

Heart failure is characterized by a complex interplay of impaired cardiac function, altered fluid homeostasis, and neurohormonal activation.^1^, ^2^ Effective management of volume status is critical to improve symptoms and outcomes in patients with heart failure.^3^ Sodium–glucose co‐transporter 2 (SGLT2) inhibitors have become a cornerstone in the management of chronic heart failure (CHF), showing significant cardiovascular benefits that are independent of glycaemic control.^4^, ^5^, ^6^ Empagliflozin, a selective SGLT2 inhibitor, has demonstrated substantial reductions in heart failure hospitalizations and cardiovascular mortality in large randomized clinical trials.^7^, ^8^ Interestingly, the pathophysiological milieu of heart failure and diabetes mellitus is associated with an up‐regulation of SGLT2 expression in proximal renal tubules, thereby supporting the rationale for SGLT2 inhibitors as a targeted therapeutic approach.^9^, ^10^, ^11^ Beyond their natriuretic and osmotic diuretic effects, SGLT2 inhibitors appear to exert beneficial haemodynamic and neurohormonal effects, although the underlying mechanisms are not fully understood.^12^

The initiation of SGLT2 inhibition is known to induce osmotic diuresis and natriuresis, which may contribute to early decongestive effects and improved haemodynamic status.^13^ However, studies suggest that these effects are modest, transient, and not sustained over time.^14^, ^15^, ^16^ While acute natriuretic and diuretic responses to SGLT2 inhibitors have been described, the durability of these effects and the body's compensatory mechanisms to preserve sodium and water balance during chronic therapy are not fully elucidated. Moreover, the activation of vasopressin‐mediated water conservation pathways in response to glucosuria‐induced osmotic diuresis remains a subject of growing interest. To address these knowledge gaps, we aimed to characterize both the renal and neurohormonal adaptations associated with SGLT2 inhibition in patients with CHF.

## Methods

### Trial design

This is a secondary analysis of a prospective, investigator‐initiated, double‐blind, placebo‐controlled study conducted at the Clinical Research Centre of the University Hospital Erlangen, Germany (NCT03128528). We enrolled patients with heart failure with reduced (HFrEF) or mildly reduced ejection fraction (HFmrEF) (as defined by 2016 European Society of Cardiology guidelines) who were in stable condition with New York Heart Association (NYHA) class II–III. The study protocol began with a 2‐week run‐in phase for each patient, allowing for medication adjustments and additional diagnostic testing as needed. Eligible patients were required to be either SGLT2 inhibitor‐naïve or off any SGLT2 inhibitor treatment for at least 10 weeks. All patients underwent a ^23^Na‐magnetic resonance imaging (MRI) examination of the lower leg at baseline and a comprehensive clinical assessment. Participants were then randomized in a 2:1 ratio to receive either empagliflozin 10 mg daily (*n* = 48) or placebo (*n* = 26) for 3 months. Follow‐up assessments, including ^23^Na‐MRI parameters and clinical parameters, were conducted at 1 and 3 months. The evaluation process of the efficacy parameters was fully blinded, with investigators unaware of clinical data, treatment allocation, and timing of measurements.

### Trial population

The study included patients aged 18 to 85 years with CHF who had either reduced ejection fraction (HFrEF) below 40% or mildly reduced ejection fraction (HFmrEF) between 40 and 49%, accompanied by elevated N‐terminal pro‐B‐type natriuretic peptide (NT‐proBNP) levels above 125 pg/ml and at least one structural abnormality of the left atrium or ventricle. All patients were required to be in a stable clinical condition. We excluded patients with any form of diabetes other than type 2 diabetes, those receiving insulin therapy or more than one oral anti‐diabetic medication, and those who had used SGLT2 inhibitors within 10 weeks before screening. Additional exclusion criteria included treatment with high‐dose loop diuretics (defined as furosemide >80 mg/day, torasemide >40 mg/day, or piretanide >6 mg/day), uncontrolled diabetes (defined as glycated haemoglobin ≥10% or fasting plasma glucose >240 mg/dl), uncontrolled blood pressure (≥ 180/100 mmHg), severe renal impairment (estimated glomerular filtration rate [eGFR] <30 ml/min/1.73 m^2^), or NYHA class IV heart failure within the previous 3 months, and MRI contraindications.

### Clinical parameters

We collected detailed demographic data at the initial patient visit. The randomization visit included a comprehensive clinical assessment, with fasting blood samples collected to measure NT‐proBNP levels, serum sodium, serum potassium, serum urea, fasting plasma glucose, and key safety parameters including serum creatinine and eGFR. In addition, plasma copeptin concentrations were measured as a stable and reliable surrogate biomarker of vasopressin (antidiuretic hormone), given its established role in accurately reflecting endogenous vasopressin secretion and its pathophysiological relevance in regulating fluid homeostasis and osmotic balance.^17^ In parallel, 24‐h urine samples were collected using standardized protocols to ensure complete and accurate urine sampling. Urine volume, sodium, and glucose excretion were assessed. After a standardized 5‐min rest period, we measured office blood pressure and heart rate. All parameters were assessed at baseline (visit 1), after 1 month (short‐term; visit 2), and after 3 months (mid‐term; visit 3) of treatment. We documented all adverse events at each study visit.

### Urinary parameters and calculations

For each study visit, participants were instructed to bring a complete 24‐h urine collection from the preceding day. Sodium and glucose excretion were calculated from 24‐h urine collections by multiplying the measured urinary concentrations by the total urine volume. Specifically: uNaV (mmol/day) = [Na^+^] × Uvol; uGluV (mmol/day) = [Glucose] × Uvol, where [Na^+^] and [Glucose] represent urinary sodium and glucose concentrations (in mmol/L), and Uvol is the 24‐h urine volume (in liters). Estimated urinary osmolality (eUOsm) and plasma osmolality (ePOsm) were calculated based on the concentrations of key solutes using the following formula: eOsm (mOsm/kg) = (2 × [Na^+^]) + (2 × [K^+^]) + [Urea] + [Glucose] (all concentrations in mmol/L). Free water clearance (FWC) was calculated to assess renal water handling using the formula: FWC (ml/day) = UVol × (1 − eUOsm/ePOsm).

### ^23^Na‐MRI measurements

In all trial participants, tissue sodium content in the lower leg was measured with a 3.0 T clinical magnetic resonance system (Magnetom Skyra, Siemens Healthineers, Erlangen, Germany) using a custom‐made transmit/receive sodium RF birdcage knee coil (32.6 MHz, Stark Contrast, Erlangen, Germany). This method has been validated to reliably quantify tissue sodium concentrations *in vivo* in humans, with evidence from studies in human amputees demonstrating a strong correlation between sodium MRI measurements and chemically analysed tissue sodium content.^18^

### Statistical analysis

Statistical analyses were conducted using SPSS Statistics version 29 (IBM Corp., Armonk, NY, USA). Continuous variables are reported as mean ± standard deviation for normally distributed data. Continuous variables were compared using the appropriate parametric or non‐parametric tests, depending on data distribution. For categorical variables, the Chi‐square test was applied. Data from ^23^Na‐MRI, along with clinical and laboratory characteristics, were analysed using Student's *t*‐test for paired samples (baseline vs. 1 or 3 months) and unpaired samples (empagliflozin vs. placebo), provided the data followed a normal distribution.

## Results

### Baseline characteristics

The baseline characteristics of the study population are summarized in *Table* *1*. The cohort consisted of 74 patients diagnosed with heart failure. The mean patient age was 66.4 years, 84% were male, the mean ejection fraction was 40%, and the mean NT‐proBNP was 707.9 pg/ml. All patients were receiving appropriate treatment for heart failure with no change to the therapy regimen after the randomization visit until the end of the trial period. At baseline, patients were clinically stable, classified as NYHA functional class II–III, and exhibited no clinical signs of congestion, including macro‐oedema, orthopnoea, pulmonary crackles, or wheezing.

**Table 1:** Clinical characteristics of the study cohort at the baseline

Clinical characteristics	All (*n* = 74)	Empagliflozin (*n* = 48)	Placebo (*n* = 26)	*p*‐value
Demographic data				
Age (years)	66.4 ± 8.9	67.7 ± 8.8	64.0 ± 8.8	0.076
Sex (male/female)	62/12	39/9	23/3	0.522
Weight (kg)	89.0 ± 13.5	88.0 ± 13.5	90.9 ± 16.2	0.382
Body mass index (kg/m^2^)	29.0 ± 3.8	28.8 ± 3.9	29.5 ± 3.5	0.401
Cardiovascular history				
Left ventricular ejection fraction (%)	40 (35–45)	43 (37–45)	38 (28–43)	0.060
HFmrEF, *n* (%)	44 (59.5)	32 (67)	12 (46)	
HFrEF, *n* (%)	30 (40.5)	16 (33)	14 (54)	
Cause of heart failure, *n* (%)				
Ischaemic	52 (70)	33 (69)	19 (73)	
Non‐ischaemic	22 (30)	15 (31)	7 (27)	
Comorbidities, *n* (%)				
Type 2 diabetes	17 (23)	12 (25)	5 (19)	0.573
Arterial hypertension	57 (77)	35 (73)	22 (85)	0.253
Atrial fibrillation	32 (43)	21 (44)	11 (42)	0.905
Office BP, mmHg				
Systolic BP	122.2 ± 18.7	122.8 ± 19.7	121.0 ± 17.1	0.707
Diastolic BP	89.4 ± 13.1	84.4 ± 13.4	91.4 ± 12.5	0.931
Office heart rate (bpm)	68.5 ± 12.7	68.5 ± 14.4	68.6 ± 12.0	0.960
Laboratory values				
NT‐proBNP (pg/ml)	707.9 ± 638.6	733.1 ± 654.7	662.3 ± 618.4	0.653
HbA1c (%)	5.9 ± 0.6	5.9 ± 0.8	5.9 ± 0.7	0.954
Fasting plasma glucose (mg/dl)	101.5 ± 17.1	101.2 ± 15.1	102.0 ± 20.5	0.856
Serum creatinine (mg/ml)	1.04 ± 0.25	1.02 ± 0.23	1.09 ± 0.29	0.225
eGFR (ml/min/1.73 m^2^)	73.5 ± 17.6	74.1 ± 16.3	72.6 ± 19.9	0.731
Serum triglyceride (mg/dl)	132.5 ± 76.8	135.6 ± 84.7	126.7 ± 60.7	0.636
Serum cholesterol (mg/dl)	177.9 ± 45.2	176.1 ± 48.0	181.3 ± 40.1	0.636
Serum LDL‐cholesterol (mg/dl)	113.0 ± 35.0	110.9 ± 36.5	117.0 ± 32.2	0.476
Serum HDL‐cholesterol (mg/dl)	46.5 ± 11.0	46.8 ± 11.3	45.9 ± 10.5	0.736
Haemoglobin (g/dl)	13.7 ± 1.2	13.6 ± 1.2	13.8 ± 1.1	0.635
Haematocrit (%)	40.7 ± 3.5	40.6 ± 3.8	41.0 ± 2.8	0.669
Urinary albumin‐to‐creatinine ratio (mg/g)	58.2 ± 89.4	62.4 ± 108.2	51.3 ± 49.3	0.709
Heart failure treatment, *n* (%)				
ACE‐inhibitors	35 (47)	19 (40)	16 (62)	0.071
AT1‐receptor antagonists	20 (27)	15 (31)	5 (19)	0.266
Sacubitril/valsartan	12 (16)	5 (10)	7 (30)	0.743
Calcium channel blocker	13 (18)	8 (17)	5 (19)	0.760
Mineralocorticoid receptor antagonist	41 (55)	24 (50)	17 (65)	0.204
Thiazide	15 (20)	8 (17)	7 (27)	0.295
Loop diuretic	29 (40)	19 (40)	10 (38)	0.972

ACE, angiotensin‐converting enzyme; AT1, angiotensin II type 1; BP, blood pressure; eGFR, estimated glomerular filtration; HbA1c, glycated haemoglobin; HDL, high‐density lipoprotein; HFmrEF, heart failure with mildly reduced ejection fraction; HFrEF, heart failure with reduced ejection fraction; LDL, low‐density lipoprotein; NT‐proBNP, N‐terminal pro‐B‐type natriuretic peptide.

### Sodium and fluid balance

Treatment with empagliflozin led to significant changes in urinary parameters and skin sodium content over the 3‐month follow‐up period (*Table* *2*). In patients treated with empagliflozin, natriuresis increased significantly during the first month (*p* = 0.040) of treatment compared to baseline, though this increase was not statistically significant when compared to the placebo group. Natriuresis returned to baseline levels by month 3 (*p* = 0.190).

**Table 2:** Longitudinal changes in markers of water and electrolyte handling during 3‐month treatment with empagliflozin versus placebo

Visit	Placebo (*n* = 26)	Empagliflozin (*n* = 48)	*p*‐value (placebo vs. empagliflozin)	*p*‐value visit 1 vs. 2 visit 1 vs. 3 (placebo)	*p*‐value visit 1 vs. 2 visit 1 vs. 3 (empagliflozin)
Body weight (kg)					
1	90.9 ± 13.4	88.0 ± 13.5	0.382		
2	90.2 ± 13.4	87.6 ± 13.7	0.465	0.369	**0.039**
3	90.5 ± 13.6	87.1 ± 13.4	0.322	0.310	**0.002**
Haematocrit (%)					
1	41.0 ± 2.8	40.6 ± 3.8	0.669		
2	41.0 ± 3.3	41.3 ± 3.8	0.741	0.978	0.055
3	41.6 ± 3.2	42.8 ± 3.9	0.196	0.225	**<0.001**
Fasting blood glucose (mmol/L)					
1	5.7 ± 1.1	5.6 ± 0.8	0.856		
2	5.9 ± 1.7	5.4 ± 0.7	0.103	0.090	**0.005**
3	5.6 ± 1.2	5.3 ± 0.8	0.170	0.981	**<0.001**
Urine volume (ml/day)					
1	1826.9 ± 755.5	1867.7 ± 672.9	0.812		
2	1911.7 ± 671.1	2261.3 ± 717.5	0.050	0.548	**<0.001**
3	1914.2 ± 740.6	2089.6 ± 640.6	0.302	0.649	**0.019**
eUOsmo (mOsm/kg H_2_O)					
1	521.4 ± 150.9	459.3 ± 184.0	0.152		
2	493.7 ± 145.6	488.5 ± 150.7	0.890	0.383	0.105
3	462.0 ± 129.9	499.4 ± 157.7	0.320	0.074	**0.019**
FWC (ml/day)					
1	−1263.8 ± 665.5	−873.5 ± 949.7	0.067		
2	−1249.8 ± 960.3	−1420.1 ± 1094.9	0.463	0.922	**<0.001**
3	−1009.5 ± 921.2	−1397.9 ± 1102.4	0.132	0.124	**<0.001**
UNaV (mmol/day)					
1	182.7 ± 85.3	161.5 ± 61.6	0.225		
2	187.2 ± 89.1	186.6 ± 79.4	0.976	0.854	**0.040**
3	175.3 ± 82.2	177.6 ± 81.5	0.909	0.626	0.190
UGlucV (mmol/day)					
1	2.8 ± 11.2	1.1 ± 4.2	0.366		
2	11.9 ± 53.3	190.7 ± 112.3	**<0.001**	0.309	**<0.001**
3	1.1 ± 4.0	198.0 ± 124.0	**<0.001**	0.251	**<0.001**
Skin sodium content (AU)					
1	21.3 ± 5.0	22.8 ± 6.1	0.367		
2	20.9 ± 4.9	21.6 ± 6.0	0.587	0.658	**0.039**
3	22.3 ± 5.8	21.5 ± 6.0	0.627	0.384	**0.013**
Muscle sodium content (AU)					
1	18.6 ± 3.9	19.5 ± 3.4	0.380		
2	17.5 ± 2.6	18.8 ± 3.3	0.108	0.061	0.138
3	18.2 ± 3.1	19.3 ± 3.7	0.227	0.169	0.448

Data are presented as mean ± standard deviation.

AU, arbitrary units; eUOsmo, estimated urine osmolality; FWC, free water clearance; UGlucV, 24‐h urine glucose excretion; UnaV, 24‐h urine sodium excretion; Visit 1, baseline; Visit 2, after 1 month of treatment; Visit 3, after 3 months of treatment.

Similarly, skin sodium content showed a significant reduction at 1 month (*p* = 0.039) compared to baseline and maintained this reduction at 3 months (*p* = 0.013). Moreover, the 3‐month reduction in skin sodium content was also statistically significant when compared to the placebo group (*p* = 0.022), indicating a sustained effect of empagliflozin on tissue sodium content.

Glucosuria persisted throughout the entire study duration (all time‐points: *p* < 0.001), contributing to a significant increase in estimated urinary osmolality in the empagliflozin group compared to placebo at the 3‐month follow‐up (*p* = 0.003). Urine volume also increased at 1‐month follow‐up (*p* = 0.046), but normalized by month 3, showing no significant difference from the placebo group. Patients receiving empagliflozin exhibited a significant reduction in FWC at both 1‐month (*p* = 0.008) and 3‐month (*p* < 0.001) follow‐up visits. There was a modest but significant decrease in body weight (*p* = 0.010). Serum sodium, potassium, and urea concentrations remained stable throughout the study period. Haematocrit levels increased significantly in the empagliflozin group after 3 months compared to placebo (*p* = 0.002).

### Adaptive neurohormonal mechanisms—copeptin response

Copeptin concentrations were measured to investigate the alterations in water balance and to characterize both early and mid‐term neurohormonal adaptations to empagliflozin therapy (*Table* *3*). Subjects receiving empagliflozin showed significant elevations in copeptin levels of 3.6 ± 5.8 pmol/L at 1 month (*p* < 0.001) and 2.6 ± 4.6 pmol/L at 3 months (*p* < 0.001) after treatment initiation. In contrast, no significant changes were observed in the placebo group. Moreover, the changes in copeptin levels were significantly greater in the empagliflozin group than in the placebo group at both 1 month (*p* = 0.020) and 3 months (*p* = 0.001), suggesting a sustained neurohormonal response to the treatment (*Table* *4*).

**Table 3:** Copeptin levels at baseline, after 1 month, and 3 months of treatment with empagliflozin or placebo

	Copeptin at baseline (pmol/L)	Copeptin at 1‐month follow‐up (pmol/L)	*p*‐value (baseline vs. 1 month)	Copeptin at 3‐month follow‐up (pmol/L)	*p*‐value (baseline vs. 3 months)
All (*n* = 74)	10.9 ± 11.4	13.2 ± 10.5	**0.004**	12.0 ± 14.0	0.292
Empagliflozin (*n* = 48)	9.5 ± 7.6	13.1 ± 9.5	**<0.001**	12.4 ± 8.3	**<0.001**
Placebo (*n* = 24)	13.8 ± 16.3	13.5 ± 12.4	0.874	11.3 ± 10.3	0.145

Data are presented as mean ± standard deviation.

**Table 4:** Changes in sodium balance and fluid regulation after 1 month and 3 months of treatment with empagliflozin or placebo

	Empagliflozin (change 1 month from baseline)	Placebo (change 1 month from baseline)	*p*‐value (empagliflozin vs. placebo 1‐month follow‐up)	Empagliflozin (change 3 months from baseline)	Placebo (change 3 months from baseline)	*p*‐value (empagliflozin vs. placebo 3‐month follow‐up)
Body weight (kg)	−0.4 ± 1.4	0.3 ± 1.5	0.053	−0.8 ± 1.8	0.5 ± 2.3	**0.010**
Haematocrit(%)	0.7 ± 2.5	−0.01 ± 2.2	0.233	2.2 ± 2.3	0.5 ± 1.8	**0.002**
Fasting blood glucose (mmol/L)	−3.9 ± 9.2	4.3 ± 11.8	**0.002**	−6.6 ± 9.2	0.1 ± 8.7	**0.005**
Urine volume (ml/day)	393.5 ± 643.4	74.2 ± 596.5	**0.046**	221.9 ± 634.2	76.7 ± 814.5	0.409
eUOsmo (mOsm/kg H_2_O)	28.2 ± 115.6	−25.0 ± 134.7	0.093	40.4 ± 112.7	−57.6 ± 147.5	**0.003**
FWC (ml/day)	−546.6 ± 808.6	14.0 ± 676.1	**0.006**	−530.4 ± 868.9	238.5 ± 697.7	**<0.001**
UNaV (mmol/day)	25.2 ± 81.8	2.8 ± 73.4	0.263	16.0 ± 82.5	−9.1 ± 90.5	0.244
UGlucV (mmol/day)	189.5 ± 112.7	9.3 ± 43.0	**<0.001**	196.8 ± 124.4	−1.9 ± 7.6	**<0.001**
Copeptin (pmol/L)	3.6 ± 5.8	−0.3 ± 7.7	**0.020**	2.6 ± 4.6	−2.6 ± 4.6	**0.001**
Skin sodium content (AU)	−1.2 ± 3.9	−0.4 ± 3.5	0.411	−1.3 ± 3.5	0.8 ± 3.5	**0.022**
Muscle sodium content (AU)	−0.7 ± 3.2	−1.1 ± 2.6	0.664	−0.3 ± 2.9	−0.8 ± 3.0	0.536

Data are presented as mean ± standard deviation.

AU, arbitrary units; eUOsmo, estimated urine osmolality; FWC, free water clearance; UGlucV, 24‐h urine glucose excretion; UnaV, 24‐h urine sodium excretion.

## Discussion

This study reveals new insights into sodium balance shifts, fluid regulation, and neurohormonal adaptations that occur during SGLT2 inhibitor treatment in patients with CHF. We observed a transient increase in natriuresis during the first month of empagliflozin treatment, which subsequently returned to baseline by 3 months, consistent with prior reports suggesting that the natriuretic effect of SGLT2 inhibitors is self‐limiting.^19^, ^20^ This suggests the presence of compensatory renal adaptations that mitigate the sustained loss of sodium, likely due to counter‐regulatory mechanisms such as enhanced sodium reabsorption in the downstream segments of the nephron.^11^ Despite the normalization of urinary sodium excretion, skin sodium content remained significantly reduced throughout the follow‐up period, suggesting a sustained effect of empagliflozin on tissue sodium content (*Graphical Abstract*). This may reflect a beneficial shift in sodium redistribution not captured by urinary output alone and is consistent with previous evidence indicating that SGLT2 inhibition modulates non‐osmotic sodium storage mechanisms.^21^, ^22^ A reduced tissue sodium content may lead to an attenuated hypertrophic signalling, e.g. diminishing directly hypertrophic responses in myocardial myoblasts and vascular smooth muscle cells according to studies conducted in neonatal rats.^23^ Furthermore, in human skin sodium content of the lower leg closely modulated the extent of the left ventricular hypertrophy, independent of 24‐h ambulatory blood pressure.^24^

Persistent glucosuria throughout the study period was associated with a significant increase in estimated urine osmolality at 3 months after treatment. A reduction in FWC was observed in the empagliflozin group at both 1 month and 3 months, suggesting a shift in renal water handling toward conservation in response to ongoing osmotic diuresis. Despite this change, serum sodium, potassium, and urea levels remained stable.

As previously demonstrated in other studies, haematocrit levels increased significantly in patients receiving empagliflozin therapy, and we observed a similarly significant rise after 3 months compared to placebo. This finding is consistent with the notion that SGLT2 inhibition induces an initial contraction of plasma volume, followed over time by stimulation of erythropoiesis, likely mediated through increased erythropoietin production.^25^, ^26^ The observed rise in haematocrit thus offers further evidence that the osmotic diuresis induced by empagliflozin triggers a coordinated and physiologically effective plasma volume adaptation.

In parallel, we observed a significant increase in copeptin concentrations, an established surrogate marker of vasopressin activity, in the empagliflozin‐treated group at both 1 month and 3 months. This suggests early and sustained activation of the vasopressin (antidiuretic) system as a counter‐regulatory response to the osmotic diuresis and reduced FWC effected by empagliflozin. Previous studies have documented elevated copeptin and vasopressin concentrations during SGLT2 inhibitor therapy in diabetic patients and those with chronic kidney disease.^27^ In heart failure patients specifically, Marton *et al*.^28^ reported increased copeptin levels with dapagliflozin treatment at 48 h and 4 weeks, similar to our observations. However, our study extends these findings by demonstrating that vasopressin activation persists at 3 months of continuous SGLT2 inhibitor therapy. This suggests that the water conservation response represents a sustained physiological adaptation rather than a transient compensatory mechanism.

Importantly, the observed copeptin increase occurred without significant alterations in serum sodium or clinical signs of dehydration, supporting the interpretation that this response was physiologic rather than maladaptive. Taken together, our findings support the hypothesis that the renal effects of empagliflozin are modulated over time by adaptive mechanisms involving vasopressin‐mediated water conservation. These adaptations may contribute to the favourable safety profile of SGLT2 inhibitors in heart failure and help explain how volume homeostasis is preserved despite ongoing glucosuria.

### Limitations

This study has several limitations that should be considered when interpreting the findings. First, it was a single‐centre trial with a relatively small sample size (*n* = 74). Additionally, the study population was restricted to patients with HFrEF or HFmrEF and did not include patients with heart failure with preserved ejection fraction (HFpEF), who represent a substantial proportion of the heart failure population. The strict inclusion and exclusion criteria, excluding patients with advanced diabetes, high‐dose diuretics, or severe renal impairment, limit the applicability of the results to a broader heart failure population. The follow‐up period was limited to 3 months, allowing assessment of short‐ and intermediate‐term effects but not long‐term adaptations. Future studies should aim to confirm these findings in larger, more diverse cohorts, including patients with HFpEF, and incorporate longer‐term follow‐up to better characterize the durability of the observed effect.

## Conclusion

Empagliflozin induces a transient but not sustained natriuretic effect in patients with CHF, whereas skin sodium content remains reduced. These findings suggest that the reduced skin sodium content was mediated without counter‐regulatory processes. Glucosuria and subsequent osmotic diuresis were evident after 1 and 3 months compared to baseline. Compensatory, FWC was reduced due to the increase in vasopressin to maintain plasma osmolality. Osmotic diuresis and compensatory down‐regulation of FWC maintained euvolaemia in CHF patients, indicating the kidneys' adaptive response to preserve volume homeostasis.
